# Survival and growth of a high-mountain daisy transplanted outside its local range, and implications for climate-induced distribution shifts

**DOI:** 10.1093/aobpla/plac014

**Published:** 2022-03-24

**Authors:** Emma E Sumner, John W Morgan, Susanna E Venn, James S Camac

**Affiliations:** 1 Research Centre for Applied Alpine Ecology, La Trobe University, Bundoora, VIC 3086, Australia; 2 Department of Ecology, Environment and Evolution, La Trobe University, Bundoora, VIC 3086, Australia; 3 Centre for Integrative Ecology, Deakin University, Burwood, VIC 3125, Australia; 4 Centre of Excellence for Biosecurity Risk Analysis, The University of Melbourne, Parkville, VIC 3010, Australia

**Keywords:** Biotic interactions, climate change, fundamental niche, species distribution, stress-gradient hypothesis, transplant experiment, vital rates

## Abstract

Field transplant experiments can improve our understanding of the effects of climate on distributions of plants versus a milieu of biotic factors which may be mediated by climate. We use a transplant experiment to test how survival and growth of a mountain-top daisy (*Podolepis robusta*), when planted within and outside its current local range, varies as a function of individual plant size, elevation, aspect and the presence of other vegetation. We expected a home-site advantage for the species, with highest survival and growth within the species’ current elevational limits, and a decline in vital rates above (due to physiological limitations) and below (due to competition with near-neighbours) these limits. Transplant survival during the beginning of the census was high (89 %), though by the third growing season, 36 % of initial transplants were remaining. Elevation had a significant negative effect on individual mortality rates; plants growing at higher elevations had a lower estimated hazard rate and thus, higher survival relative to those planted at elevations below the current lower limit of the distribution. By contrast, we detected no significant effect of elevation on growth rates. Small vegetation gaps had no effect on growth rates, though we found a negative, but non-significant, effect on mortality rates. Aspect had a very strong impact on growth. Plants transplanted to cool aspects had a significantly lower growth rate relative to transplants growing on a warm aspect. Conversely, aspect was not a significant predictor of individual mortality rates. Restrictions on the local distribution of *P. robusta* appear to be governed by mortality drivers at lower elevation and by growth drivers associated with aspect. We highlight that our ability to understand the drivers of distributions in current and future climates will be limited if contextual- and individual-level plant responses remain understudied.

## Teaser

Field transplant experiments can improve our understanding of the effects of climate on the distributions of plants versus a milieu of biotic factors which may be mediated by climate. We use a transplant experiment to test how survival and growth of a mountain-top daisy (*Podolepis robusta*), when planted within and outside its current local range, varies as a function of individual plant size, elevation, aspect and the presence of other vegetation. Results indicate that restrictions on the local distribution of *P. robusta*appear to be governed by mortality drivers at lower elevation and by growth drivers associated with aspect.

## Introduction

Range limits of high-mountain plant species are commonly thought to arise from physiological climatic limits, particularly that of temperature (Körner 2003). However, non-climatic processes, such as the strength of biotic relationships among species, have also been identified as a critical factor governing geographical limits in mountain ecosystems (Callaway *et al.* 2002; Alexander *et al.* 2016). Whether range limits arise directly from climatic factors (i.e. physiological tolerance limits) or indirectly (i.e. species interactions), or as a function of both, remains poorly understood (Guisan and Thuiller 2005; Warren and Bradford 2011; Moyes *et al.* 2015; Crepaz *et al.* 2021), but the interplay between abiotic and biotic drivers will be important when assessing the ecological consequences of environmental changes (Ehrlen and Morris 2015; Kadowaki *et al.* 2016; Westoby *et al.* 2017; Rayfield *et al.* 2021; Usinowicz and Levine 2021).

The consequences of accelerated climate change will be most evident at the edges of a species’ distribution where range expansions (leading edge) or contractions (trailing edge) may occur (Cahill *et al.* 2014). Commonly, species climatic envelopes (realized niches in environmental space) have been estimated and projected into the future using species distribution models based on occurrence records or various physiological climatic limits (Hirzel *et al.* 2002; Parolo *et al.* 2008; Austin and Van Niel 2011). Increasingly, such models incorporate non-climatic processes that contribute to a species’ distribution (e.g. competition, disturbance) via their impact on species’ vital rates (Parolo *et al.* 2008; Eskildsen *et al.* 2013; Briscoe *et al.* 2019). But there remain limits to this understanding, particularly with respect to plant regeneration, a critical stage in the maintenance of plant populations and their expansion (Grubb 1977; Warren and Bradford 2011). This may lead to uncertainty about predicting range shifts at local rather than large scales (Pollock *et al.* 2014).

It is at local scales where immediate factors, whether they are edaphic, microclimatic or biotic, play a role in setting species current and future distributions (Körner 2003; Niittynen *et al.* 2020; Crepaz *et al.* 2021). Interactions with competitors, for example, can strongly influence local distributions, and studies of natural range expansions point to the potential for competition from resident species to reduce expansion rates (Worth *et al.* 2017; Crepaz *et al.* 2021). However, there are too few studies that assess how these processes operate, or whether these interactions will impede or accelerate climate change-induced range shifts (HilleRisLambers *et al.* 2013, Camac *et al.* 2015, 2017; Rayfield *et al.* 2021; Usinowicz and Levine 2021) to robustly generalize on their importance.

Field transplant experiments across local range boundaries are therefore urgently required. We still know very little about which boundaries are set by competition rather than by direct effects of temperature or other environmental variables (Hargreaves *et al.* 2014). Reliable knowledge about this can come from transplanting species beyond the boundary, with and without competitors (i.e. the ‘brute force’ direct experiments advocated by Holt 2009). Field transplant experiments are the most important tool for understanding species movements under global warming (van der Veken *et al.* 2012; HilleRisLambers *et al.* 2013; Hargreaves *et al.* 2014; Kadowaki *et al.* 2016). Such experiments can elucidate how vital rates within a species’ distribution vary, particularly at their limits. Vital rates—such as growth and survival—are likely to be higher at the core of a species’ distribution and decrease closer to the boundary, eventually becoming so low that a population cannot persist beyond a particular climate limit. Without appropriate interaction estimates, we limit our ability to predict future rates of community change, the mechanisms that drive such change and how warming effects vary across landscapes (Usinowicz and Levine 2021). To accurately predict future species distributions in a changing climate, it is therefore imperative we understand how species respond to the tightly linked abiotic and biotic factors controlling seedling establishment (Alexander *et al.* 2015; Frei *et al.* 2018).

Here, we investigate the role of plant interactions and climate factors on growth and survival of a species within and outside its current mountain-top range. It is difficult to elucidate mechanisms underlying variation in vital rates, primarily due to the difficulty of obtaining detailed long-term data sets from natural plant communities. Hence, we employ a manipulative field transplant experiment with the high-mountain daisy *Podolepis robusta* to examine how climate drivers (temperature, as influenced by elevation and aspect) affect early seedling survival and growth in the presence/absence of near-neighbours. We predict that both climate and biotic interactions affect vital rates, but in different ways across the local range (Choler *et al.* 2001). During the early life-history stages, plants are especially susceptible to the abiotic conditions present in high-mountain environments (Venn *et al.* 2009; Briceño *et al.* 2015). At high elevations, facilitative interactions between close neighbouring plants may buffer seedlings from these abiotic pressures by providing shelter from frosts and winds. At lower elevations, seedlings may not be so limited by the abiotic environment, and may therefore compete for resources with close neighbouring plants (i.e. the ‘stress-gradient hypothesis’; Maestre *et al.* 2009). We use an elevational gradient transplant study to understand the controls on the local distribution of *P. robusta* (particularly outside the current local range) to inform how the species’ local distribution may change with climate warming.

## Methods

### Study species

We followed the vital rates of *P. robusta* (Asteraceae), a non-clonal, tufted perennial herb (10–40 cm tall) of subalpine forests and alpine grasslands in the Australian Alps (Costin *et al.* 2000). Plants have a thickened rootstock from which they grow leaves annually after snowmelt. Flowering of mature plants occurs annually in mid-summer. Seed dispersal is short-distance; mean seed mass is 0.97 mg and modelled maximum dispersal distance is 24.6 m (Morgan and Venn 2017). Seedling recruitment in the field is apparently rare (Venn and Morgan 2009).

### Study site

The study was conducted at Mount Hotham in south-eastern Australia (36°59ʹ24″S, 147°9ʹ0″E). The climate is cool-temperate with an average annual precipitation of 1454 mm which mostly falls as snow between June and September (Bureau of Meteorology, www.bom.gov.au). Mean maximum temperatures in the growing season range from 11.4 °C (November) to 16.6 °C (January), with mean minimum temperatures in the growing season ranging from 3.8 °C (November) to 8.2 °C (January) (Bureau of Meteorology, www.bom.gov.au). Sub-zero temperatures can occur at any time of year and are common during the growing season (McDougall and Walsh 2007). Soils are organic, acidic and are typically shallow (Costin 1954).

At Mount Hotham, *P. robusta* was restricted to north-west-facing (warm), mid-elevation slopes in the vicinity of eucalypt low forest. Relative abundance was determined through preliminary transect surveys conducted in 2012 across the study site. *Podolepis robusta* was absent below ~1700 m (‘lower elevation distribution’) and above ~1780 m (‘upper elevation distribution’). It was also never observed on south-east-facing, ‘cool’ aspects **[see**Supporting Information—**Fig. S1****]**.

### Study design

At the core of our experimental approach was a 3-year transplant experiment conducted at 10 transplant sites along a 235-m elevation gradient (1620–1855 m) to test for the effect of elevation on vital rates (Table 1). Temperature (due to the lapse rate) and soil depth decline with elevation (Körner 2003). To examine whether vital rates at each elevation vary with respect to aspect, five of the sites were established on cool, south-east-facing aspect slopes and five sites were on warm, north-west-facing aspect slopes. Slopes were similar on both opposing aspects. Aspect affects soil moisture (Isard 1986) and growing season (measured as growing degree-days), with snowpack lasting longer at high elevations on south-east-facing slopes than north-west-facing slopes (Edmonds *et al.* 2006). Frost frequency increased with elevation and was affected by aspect; frost was more frequent on south-east aspects relative to north-west aspects (Table 1).  Transplant sites were randomly selected at 30- to 110-m elevation intervals on both aspects (Table 1; see Supporting Information—Fig. S1).

**Table 1.** Location and setting of *Podolepis robusta* transplant planting sites at Mt Hotham, Australia. Sites 1–4 were ‘above’ the current local range, and Sites 6–10 were ‘below’ the current range. For each transplant site, the number of frost days (days with temperatures below 0 °C, with minimum temperature in parentheses), and growing degree-days (GDD) as recorded by temperature iButtons deployed (at 5 cm above the soil surface) during the first growing season (November 2016 to March 2017), and vegetation structure (height of densest vegetation and maximum vegetation height) are indicated.

Transplant site	Elevation (m)	Aspect (°)	Frost days	GDD	Densest vegetation height (cm)	Maximum vegetation height (cm)
1	1855	280	10 (−1.20)	1547	0–20	0–20
2	1860	115	15 (−1.28)	1490	0–20	0–20
3	1820	280	10 (−1.34)	1534	0–20	21–40
4	1800	130	12 (−1.31)	1420	0–20	0–20
5	1780	282	5 (−1.35)	1705	0–20	41–60
6	1700	270	4 (−1.35)	1544	0–20	41–60
7	1690	163	10 (−1.19)	1412	0–20	21–40
8	1670	226	0 (0.18)	1505	21–40	41–60
9	1660	146	7 (−1.31)	1593	0–20	41–60
10	1620	100	7 (−1.22)	1583	0–20	41–60

The plant community on the south-east (cool) aspect sites was typically dominated by closed heathland (to 1 m tall) at low elevations and herbfield (to 20 cm tall) at high elevations, while north-west (warm) aspect sites were dominated by heathy woodland to the treeline (1780 m), and low heathlands (to 30 cm) above the treeline.

### Determining vital rates via a transplant experiment

Seed of *P. robusta* was collected from Mt Hotham in March 2016, stored in paper bags at room temperature and surface-sown onto a mixture of sterilized perlite, sand and potting mix in October 2016. After germination, seedlings were then grown individually in 4 × 4 cm cells for 6 weeks in an unheated glasshouse, after which seedlings were acclimated at Mt Hotham for 5 days prior to planting. In mid-November 2016 (austral spring), ~4 weeks after snowmelt, seedlings (*n* = 13; mean 9.98 (± 0.17, 1 SE) leaves) were planted into either (i) intact vegetation (control, with neighbouring plants) or (ii) vegetation gaps (15 cm × 15 cm gaps where all vegetation was removed by cutting to ground level), at each of the 10 sites (*n* = 260 seedlings in total). Vegetation gaps were created only at the time of transplanting and not maintained thereafter. Gaps in the vegetation were present for much of the experiment, especially on the south-east aspect, although this was not formally quantified.

Transplanted seedlings were arranged along a transect at each site, with treatments alternating at 1-m intervals. Seedlings that did not survive after the first week of transplanting were presumed to have died from transplant shock and were immediately replaced. Following the first growing season (after ~4 months), the length of the longest living leaf was recorded to the nearest millimetre. Leaf measurements and seedling mortality were recorded in March 2017 and remeasured during the subsequent three growing seasons, until January 2020.

### Statistical analysis

In this experiment, we were interested in understanding the impacts of elevation, aspect and treatment (i.e. gap vs. control) on two fundamental demographic rates—mortality and growth.

#### Modelling seedling mortality rates.

Standard survival analysis is based on estimating an instantaneous hazard of an individual, *i*, dying at time *t*:


$$\begin{array}{l} h_{i}(t)\;=\;h_{0}\left(t\right)\;exp\;(\eta_{i}(t)) \end{array}$$

where $h_{0}(t)$ is the baseline hazard (i.e. the hazard rate for an individual when all covariates in $\eta_{i}\;$ are equal to zero) at time *t*, and $\eta_{i}(t)\;$ denotes the linear predictor evaluated for individual *i* at time *t.*

By estimating the instantaneous hazard rate, $h_{i}(t)$, we can derive the probability an individual survives to time *t* as:


$$\begin{array}{l} Pr\;(Survival_{i})\;=\;exp\left(-\int_{u=0}^{t}h_{i}(u)\;du\right) \end{array}$$

The baseline hazard, $h_{0}(t)$, can be estimated using a range of different parametric and non-parametric functions, each of which affects how the baseline hazard changes over time. In this analysis, rather than rely on a single model, we fitted three commonly used hazard functions (exponential, Weibull, Gompertz) as well as a more flexible spline-based approach.

The simplest hazard model fitted is the standard exponential hazard model which assumes that baseline hazard is constant through time:


$$\begin{array}{l} h_{i}(t)\;=\;exp(\eta_{i}(t))\; \end{array}$$

The second function fitted was the Weibull hazard function:


$$\begin{array}{l} h_{i}(t)\;=\;\gamma t^{\gamma -1}\;exp(\eta_{i}(t)) \end{array}$$

This hazard allows for the baseline hazard to monotonically increase or monotonically decrease over time. *γ* > 0 is the Weibull shape parameter. Note that when *γ* = 1, the Weibull hazard function reduces to the exponential hazard function.

The third parametric function fitted was the Gompertz hazard function:


$$\begin{array}{l} h_{i}(t)\;=\;exp(\phi t)\;exp(\eta_{i}(t))\; \end{array}$$

Here ϕ>0 is the Gompertz scale parameter. Like the Weibull hazard function, this function allows the baseline hazard to monotonically increase or decrease over time and reduces to the exponential model when ϕ=0.

The most flexible hazard function we fitted was the M-splines hazard function which attempts to directly estimate the baseline hazard using cubic M-splines such that the baseline hazard can be modelled as a flexible and smooth function of time. The underlying mechanisms of this function are outlined in Brilleman *et al.* (2020).

All four hazard functions have recently been incorporated into the Bayesian R package ‘rstanarm’ 2.21.2 (Brilleman *et al.* 2020). Here, we used the survival status of each individual in each census period coupled with this package to estimate the effect of individual size (i.e. maximum leaf length), elevation, aspect and gap treatment on the hazard rate for each individual. In order the assess these effects we defined, $\eta_{i}(t)$ as:


$$\begin{array}{l} \begin{array}{l} \eta_{i}(t)=\;\alpha +\beta_{1}\;Gap_{i}\;+\;\beta_{2\;}Leaf\;length_{i,\;t-1}\;+\;\beta_{3}\;Aspect_{i}+\;\beta_{4\;}Elevation_{i}+\\ \beta_{5}\;Aspect_{i}\;\times Gap_{i}\;+\beta_{6}\;Elevation_{i}\times Gap_{i}\;\times \beta_{7}\;Elevation_{i}\;\times \;Gap_{i}\;+\;\varepsilon_{ind[i]}+\\ \varepsilon_{site[i]} \end{array} \end{array}$$

Here, Gap is a binary predictor variable that refers to whether seedling *i* is growing within a control or Gap treatment (coded as 0 and 1, respectively). Leaf length is a continuous predictor that refers to the maximum leaf length observed at the previous census (*t* − 1), Aspect is a binary covariate that refers to whether an individual is growing on a warm NW or cool SE aspect (coded as 0 and 1, respectively) and elevation is a continuous predictor defining the elevation above sea level the seedling was growing at. Elevation and leaf size predictors were centred on 1740 m a.s.l. and 6 cm, respectively, and scaled by two times their standard deviation. This allows for comparing the magnitude of estimated effects between binary and continuous predictors (Gelman and Hill 2007). Moreover, it allows the intercept, *α*, to be interpreted at a realistic value; specifically, the predictor evaluated for a 6 cm individual growing in a control plot at 1740 m a.s.l.; the centre of the known distribution at Mt Hotham. We included three two-way interactions in order to examine whether the gap treatment effect varied with elevation or aspect, or whether the effects of aspect varied with elevation. Lastly, to account for non-independence of observations within individuals or within sites, we included individual random effects, $\varepsilon_{ind[i]}$ and site, $\varepsilon_{site[i]}$ random effects.

We applied the above linear model in all four hazard models, using default priors specified in rstanarm’s stan_surv function. Specifically, the intercept was estimated using a normal prior centred on zero with a standard deviation of 2. Predictor effects (i.e. $\beta_{1-7}$) and random effects ($\varepsilon_{ind[i]}$ and site, $\varepsilon_{site[i]}$) were estimated using normal priors centred on zero with a standard deviation of 2.5. Auxiliary parameters such as the Gompertz scale parameter, ϕ, and the Weibull shape parameter, *γ*, were estimated using half normal distributions, with both centred on zero, but with standard deviations set as 0.5 and 2, respectively. All models were run for 2000 iterations, whereby the first 1000 were discarded as burn-in. Model convergence was assessed using the Brooks–Gelman–Rubin convergence diagnostic (Brooks and Gelman 1998).

#### Modelling seedling growth rates.

We were also interested in understanding how gap treatment, elevation and aspect influenced seedling growth rates. Like hazard modelling, a large variety of parametric growth functions exist that can be used to estimate the growth rate (and trajectory) of individuals (Tjørve and Tjørve 2010; Camac *et al.* 2017). These growth functions each make different assumptions about the shape of the growth trajectory and whether growth eventually asymptotes to some maximum limit (Tjørve and Tjørve 2010).

Since leaves do not grow indefinitely, we model the growth in maximum leaf length of each individual, *i*, using three asymptotic growth functions outlined in Tjørve and Tjørve (2010):

The negative exponential growth function:


$$\begin{array}{l} Leaf\;length_{i}(t)=A\left(1\;-\left(1-\frac{leaf\;length_{i.t0}}{A}\right)\right)\;\times exp(-R_{i}t) \end{array}$$

The logistic growth function:


$$\begin{array}{l} Leaf\;length_{i}(t)=\frac{A}{\left(1+\left(\frac{A}{leaf\;length_{i,t0}}-1\right)\right)\times exp(-R_{i}t)} \end{array}$$

The von Bertalanffy growth model:


$$\begin{array}{l} Leaf\;length_{i}(t)=A\left(1+\left({\frac{leaf\;length_{i,t0}}{A}}^{\frac{1}{3}}-1\right)\right)\times exp{\left(-R_{i}t\right)}^{3} \end{array}$$

where *A* is the asymptotic maximum leaf length achievable for an individual, leaf length_*i*,*t*0_ is the initial size of individual *i* at the commencement of the experiment (*t*0), $R_{i}\;$ is the growth rate parameter for individual *i* and *t* is the time since the start of the experiment.

We fitted these three growth functions using R 4.0.3 and the Bayesian R package ‘brms’ 2.14.0 (Bürkner 2017). As negative leaf lengths are an impossibility, we modelled leaf length as a random realization from a half normal distribution, with the lower bound truncated at zero.

As our aim was to understand how gap treatment and other predictors influenced average individual growth rates, $R_{i}$, we modelled R as a function of the following log-linear model:


$$\begin{array}{l} \begin{array}{ll} log(R_{i})\;= & \alpha +\beta_{1}\;Gap_{i}\;+\;\beta_{2\;}Aspect_{i}+\;\beta_{3}\;Elevation_{i}+\beta_{4}\;Aspect_{i}\;\times Gap_{i}\;\\ & +\beta_{5}\;Aspect_{i}\;\times Elevation_{i}\;+\beta_{6}\;Gap_{i}\;\times Elevation_{i}+\;\varepsilon_{ind[i]}+\;\varepsilon_{site[i]} \end{array} \end{array}$$

In all growth models, R was modelled on the log scale to ensure growth rates were positive and that the three models produced asymptotic growth trajectories. Like the hazard models, Gap is a binary predictor variable that refers to whether the seedling *i* is growing within a control or Gap treatment (coded as 0 and 1, respectively). Aspect is a binary covariate that refers to whether an individual is growing on a warm NW or cool SE aspect (coded as 0 and 1, respectively) and elevation is a continuous predictor defining the elevation above sea level the seedling is growing at. Elevation was centred on 1740 m a.s.l. and scaled by two times their standard deviation. This allows for comparing the magnitude of estimated effects between binary and continuous predictors (Gelman and Hill 2007). Moreover, it allows the intercept, *α*, to be interpreted at a realistic value; specifically, the predictor evaluated for an individual growing in a control plot on a warm NW aspect at 1740 m a.s.l. We included three two-way interactions in order to examine whether the gap treatment effect varied with elevation or aspect, or whether the effects of aspect varied with elevation. Lastly, to account for non-independence of observations within individuals or within sites, we included individual random effects, $\varepsilon_{ind[i]}$ and site, $\varepsilon_{site[i]}$ random effects.

We fit the three growth models brms’s brm function. Predictor effects (i.e. $\beta_{1-6}$) were estimated using normal priors centred on zero with a standard deviation of 5. The average asymptotic leaf length, *A*, was estimated using a uniform prior bounded between 5 and 30 cm. Individual, $\varepsilon_{ind[i]}$, and site, $\varepsilon_{site[i]}$ random effects were estimated using Student’s *T*-distributions with three degrees of freedom, centred on zero with a standard deviation of 5.9. All models were run for 2000 iterations, whereby the first 1000 were discarded as burn-in. Model convergence was assessed using the Brooks–Gelman–Rubin convergence diagnostic (Brooks and Gelman 1998).

#### Estimating predictive accuracy and selecting model for inference.

Among hazard models and among growth models we compared the predictive accuracy using 10-fold cross-validation (Camac *et al.* 2018) whereby individuals (and their repeated observation within) were divided into 10 subsets and the model was then fit 10 times, each time using a different combination of testing (1-fold) and training (9-folds) combination. Predictive accuracy was assessed by averaging the expected log predictive density (ELPD) of the testing data set. The functional form with the mean highest ELPD was then used for model inference.

## Results

### Transplant survival

Survival of *P. robusta* transplants throughout the experiment was independent of elevation and aspect. Overall, 64 % of *P. robusta* transplants died throughout the course of this experiment. From the beginning of the census in 2017 to the end of the growing season in 2018, 24 out of 217 plants died (11 %). A further 77 deaths (39 % of 2018 cohort) were recorded in 2019, and there were 37 deaths (31 % of 2019 cohort) in 2020.

The exponential model was inferior to other hazard models; we used the M-spline model because it has a slightly higher expected mean log predicted density (ELPD) than the Gompertz and Weibull models **[see****Supporting Information—Fig. S2–S4****]**. Treatment (seedlings planted into canopy gaps) did not have a significant effect on hazard rates (Fig. 1A and B). By contrast, initial leaf length of seedlings had a significant outcome on hazard rates, with higher survival of transplants with longer leaves (Fig. 1A and C). Hazard rates were higher at low elevation (outside the current range) compared to the high elevation (above the current range; Fig. 1A and E). Aspect did not significantly affect hazard rate (Fig. 1A and D), nor were there any interactions between aspect, elevation and treatment (gaps) (Fig. 1A).

**Figure 1.** (A) Centred and standardized hazard model coefficients and the effects of (B) gap treatment, (C) leaf length, (D) aspect and (E) elevation on probability of *Podolepis robusta* transplant survival at Mt Hotham, Australia. Solid lines in panels B–E represent means. The shaded areas in panels B–E indicate 95 % credible intervals.

### Transplant growth

We used the von Bertalanffy model as our inference model as it had the highest mean ELPD, acknowledging that all three models were not statistically different (*P* > 0.05; see Supporting Information—Fig. S5–S7). Median and maximum recorded leaf lengths were 5 and 24.5 cm, respectively. There was a marginal negative effect of the gap treatment on leaf length (though not significant at the 0.05 level) (Fig. 2A and B). In contrast to survival, there was a significant negative effect of SE aspect on leaf length (Fig. 2A and C) with almost no growth over the experiment on the cool aspect. Elevation had no significant effect on growth (Fig. 2A and D), nor were there any interactions between aspect, elevation and treatment (gaps) (Fig. 2A).

**Figure 2.** (A) Centred and standardized growth model coefficients and the effects of (B) gap treatment, (C) aspect and (D) elevation on the growth of *Podolepis robusta* transplants at Mt Hotham, Australia. Solid lines in panels B–D represent means. The shaded areas in panels B–D indicate 95 % credible intervals.

### Transplant flowering

By the end of the growing season in 2019, a total of six plants had flowered (out of 112 surviving plants, i.e. 5.4 %). These were all on the warm north-west-facing aspect, with two individuals within the current range and four above the current range. A total of four plants flowered in the 2020 growing season (out of 79 surviving plants; 5.1 %) and these were also all on the warm north-west-facing aspect; two flowering plants were within the current range and two above the current range.

## Discussion

Ongoing changes in global climate are altering ecological conditions for many species and the consequences of such changes are likely most evident at the edges of the geographical distribution of a species, where range expansions or contractions may occur (Harsch *et al.* 2009; Cahill *et al.* 2014; Hargreaves *et al.* 2014). This is particularly relevant to high-mountain ecosystems where the distribution of species is often attributed to thermal tolerance and/or growing-season temperatures (Körner 2012). However, we do not know how often climate limits species from establishing populations beyond their current range, and whether temperature is the main driver (Hargreaves *et al.* 2014; Moyes *et al.* 2015; Crepaz *et al.* 2021; Rayfield *et al.* 2021; Usinowicz and Levine 2021). We used a transplant experiment over 3 years to test how survival and growth of a mountain-top daisy (*P. robusta*), when planted within and outside its current local range, varies as a function of individual plant size, elevation, aspect and the presence of other vegetation. By examining putatively limiting factors (temperature, frost, vegetation cover), we observed how plants respond to key drivers of local distribution. Our results suggest that the fundamental niche distribution of *P. robusta* was larger than its current realized niche distribution. Transplants were able to establish in areas beyond the current local range limits, with survival after 3 years observed both above and (less so) below the current local distribution. The presence of near-neighbours did not affect this outcome. While tolerance to climate and other abiotic factors are often demonstrated as having strong influences on plant species (e.g. Körner and Paulsen 2004; Niittynen *et al.* 2020), our results indicate that *P. robusta* has a broader tolerance to abiotic conditions than would be assumed based on its current local distribution. Further, if the current distribution of *P. robusta* were at equilibrium, we would expect the highest survival to be within its range, with hazard rates increasing as beyond-range distance increases. Hargreaves *et al.* (2014) found that in the majority of transplant experiments they reviewed, performance declined beyond the range. We observed that transplant survival was highest 20–40 m elevation above the current upper local distribution boundary. Growth, by contrast, was higher at lower elevations (outside the current lower local distribution limits). Hence, there was no strong evidence that there was a home-site advantage.

We focused on small transplants (planted when ~2 months old) to test for controls on survival and found that larger plants (those that grew longer leaves over the experiment) had higher survival over time than those that were smaller. Early seedling survival is thought to be a critical stage in plant population growth (Grubb 1977; Harper 1977) yet it is commonly overlooked when coupling plant distributions with environmental variables (Warren and Bradford 2011; Davis *et al.* 2016). It is at the (very) local scale where range limit factors play an important role in setting range boundaries. The interaction between recruitment and seedling mortality drivers is in need of better understanding (Sexton *et al.* 2009; Normand *et al.* 2014; Stevens-Rumann *et al.* 2018). There are too few studies that assess how or when these interactions impede or accelerate climate change-induced range shifts to robustly generalize on their importance (HilleRisLambers *et al.* 2013; Svenning *et al.* 2014; Louthan *et al.* 2015; Nooten and Hughes 2017).

We found little evidence that near-neighbours either facilitated or impeded survival and growth of seedlings of *P. robusta* at any elevation across our study mountain, although we found a marginal trend of higher growth rates when grown close to near-neighbours. This may be because growing conditions/mortality drivers in Australian high mountains are governed by factors other than temperature. Soil water shortages likely play an important role in exerting range boundaries (Pook *et al.* 1966; Griffin and Hoffmann 2012) and may explain higher hazard rates at the trailing edge. Higher evaporative demand at warmer lower elevations may exceed water availability, as was shown in the Swiss Alps (Jolly *et al.* 2005). Indeed, in mountains prone to seasonal water shortages, shifting elevational optima were better explained by altered climatic water balance rather than by temperature alone (Crimmins *et al.* 2011). Other studies carried out in drought-prone mountains have also shown that plant survival and growth is limited by water availability (Pook *et al.* 1966; Griffin and Hoffmann 2012), and that positive associations at lower elevations can be explained by the alleviation of soil water shortages by nurse plants (Cavieres *et al.* 2006).

Alternatively, regeneration processes/filters may play out at the seed germination rather than transplant survival stage (Morgan 1997; Walck *et al*. 2011). In Australian mountains, where vegetation cover is typically high, optimal gap size for regeneration may depend on life form. Williams (1992) showed that grass seedlings are favoured by bare-ground gaps <15 cm diameter while shrub seedlings are most common in bare-ground gaps >15 cm diameter. This may be due to competitive interactions between shrub seedlings and surrounding tussock grasses. Similarly, seedlings of the treeline species *Eucalyptus pauciflora* in gaps within alpine grassland suffered from competition from surrounding tussock grasses when growing within 4 cm of a tussock (Noble 1980). It is likely that competition–facilitation trade-offs vary across species, making generalizations difficult. While the bare-ground patch is integral to the regeneration of woody species (Williams 1992), it may be less important to establishment of herbaceous species such as *P. robusta*. Determining the optimal gap size requirement for herbaceous high-mountain species may help better understand their capacity to establish into new habitats as a result of climate change (Usinowicz and Levine 2021).

The survival of transplanted seedlings above the current range appears to have effectively overcome a dispersal or, more likely, an early seedling establishment barrier (Kroiss and HilleRisLambers 2015). Survival and growth of *P. robusta* was possible outside the current local range (the ‘realized niche’ *sensu*Hutchinson 1957) and indicates that factors that affect the fate of seeds and/or seedlings play an important role in local establishment. Australian alpine species, including *P. robusta*, have limited dispersal capacity (Morgan and Venn 2017) and a variable interannual phenology (Hoffmann *et al.* 2010). Pre-dispersal seed predation by insects can be high, limiting seed outputs in many years (Pickering 2009). For species responding to climate change, these factors represent non-trivial barriers to movements and establishment (Kinlan and Gaines 2003; Usinowicz and Levine 2021). While a high capacity to disperse does not necessarily equate to establishment in new locations (e.g. Alexander *et al.* 2015), we contend that short dispersal capacity is a key limitation for alpine species, like *P. robusta*, over ecological timescales.

Our study suggests that climate warming in high mountains may affect *P. robusta* not by making new habitat available for colonization *per se*, but by allowing the colonists to be more successful (where growth = success). Measuring the vital rates of individual plants over several years provided us with crucial information to assess such responses, and monitoring of individuals would ideally follow plants until they become reproductive. In our study, after three growing seasons, only ~5 % of our surviving transplants had flowered and hence, long-term demographic success (i.e. lifetime fitness) has yet to be fully realized. Co-ordinated experiments are beginning to emerge that measure vital rates outside of the current range to improve understanding of high-mountain species responses to climate warming (e.g. the global treeline expansion experiment, G-Tree; http://treelineresearch.com/). Such initiatives need to be expanded if we are to arrive at a position where we have a moderately well-founded overview of the situations where climate does, or does not, affect the range expansion of species. Can the research community collaborate to target them in such a way as to obtain generalization across species and boundary types as efficiently as possible? Agreeing to a standardized approach to experimentation has much merit for quickly advancing the field (Fraser *et al.* 2013), as has been shown by co-ordinated distributed experiments elsewhere (e.g. Nutrient Network; Borer *et al.* 2014).

## Conclusions

This study investigated the variation in vital rates of a high-mountain daisy along an elevational gradient to assess responses outside its current local range. Variations in vital rates beyond current ranges can help inform likely responses to changes in distribution drivers such as climate. Whether they are gradually displaced by novel thermophilic competitors that are able to migrate upslope, or climate niches disappear, remains contested. We show that reliable predictions of future species elevational ranges (as forecast by species distribution models) must also account for variation in microclimate afforded by topography (as it affects aspect), with survival of new seedlings and their subsequent growth likely impacted in different ways. We contend that field transplant experiments can be used to test for when a boundary will be determined by competition (and/or biotic interactions such as herbivory) versus when it will be determined by the direct effects of the physical environment. Transplant experiments are the reality-test for what sets range boundaries. Without appropriate estimates about abiotic and biotic interactions, we have very limited ability to predict future rates of change, the mechanisms that drive such change and how climate effects will vary biogeographically.

## Supporting Information

The following additional information is available in the online version of this article—

Figure S1. Study design at Mt Hotham, Australia. Ten sites were arranged along a 235-m elevation gradient, with five sites on warm, north-west-facing slopes and five sites on cool, south-east-facing slopes. *Podolepis robusta* seedlings (*n* = 13) were transplanted into either intact vegetation (control, with neighbouring plants) or vegetation gaps (15 cm × 15 cm gaps where all vegetation was removed by cutting to ground level). This figure is not representative of the total number of transplant replicates.

Figure S2. Expected log predicted density for each hazard model. The exponential model was clearly inferior to other models.

Figure S3. Unexplained residual variation among individuals derived from vb growth model.

Figure S4. Unexplained residual variation among sites derived from vb growth model.

Figure S5. Expected log predicted density for each growth model. All models were similar, though the von Bertalanffy model had slightly higher mean ELPD.

plac014_suppl_Supplementary_Figures

## Data Availability

All data and code used in this study are available at the following link: https://github.com/jscamac/Podolepis_demographics.

## Notes

**Populations & Communities. Chief Editor**: Jean Burns
