# Supplementary material for: Survival and growth of a high-mountain daisy transplanted outside its local range, and implications for climate-induced distribution shifts
Source: AoB Plants. 2022 Mar 24;14(2):plac014. doi: 10.1093/aobpla/plac014 (PMC9049260; doi:10.1093/aobpla/plac014)
Supplement: plac014_suppl_Supplementary_Figures [file plac014_suppl_supplementary_figures.pdf]

Supporting Information

Survival and growth of a high-mountain daisy transplanted outside its local range, and implication for climate-induced distribution shifts

Emma E. Sumner[1,2,3,*], **John W. Morgan**[1,2], **Susanna E. Venn**[1,3] **and James S. Camac**[1,4]

[1]Research Centre for Applied Alpine Ecology, La Trobe University, Bundoora, VIC 3086, Australia
[2]Department of Ecology, Environment and Evolution, La Trobe University, Bundoora, VIC 3086, Australia
[3]Centre for Integrative Ecology, Deakin University, Burwood, VIC 3125, Australia
[4]Centre of Excellence for Biosecurity Risk Analysis, The University of Melbourne, Parkville, VIC 3010, Australia
*Corresponding author's e-mail address: eesumner@deakin.edu.au

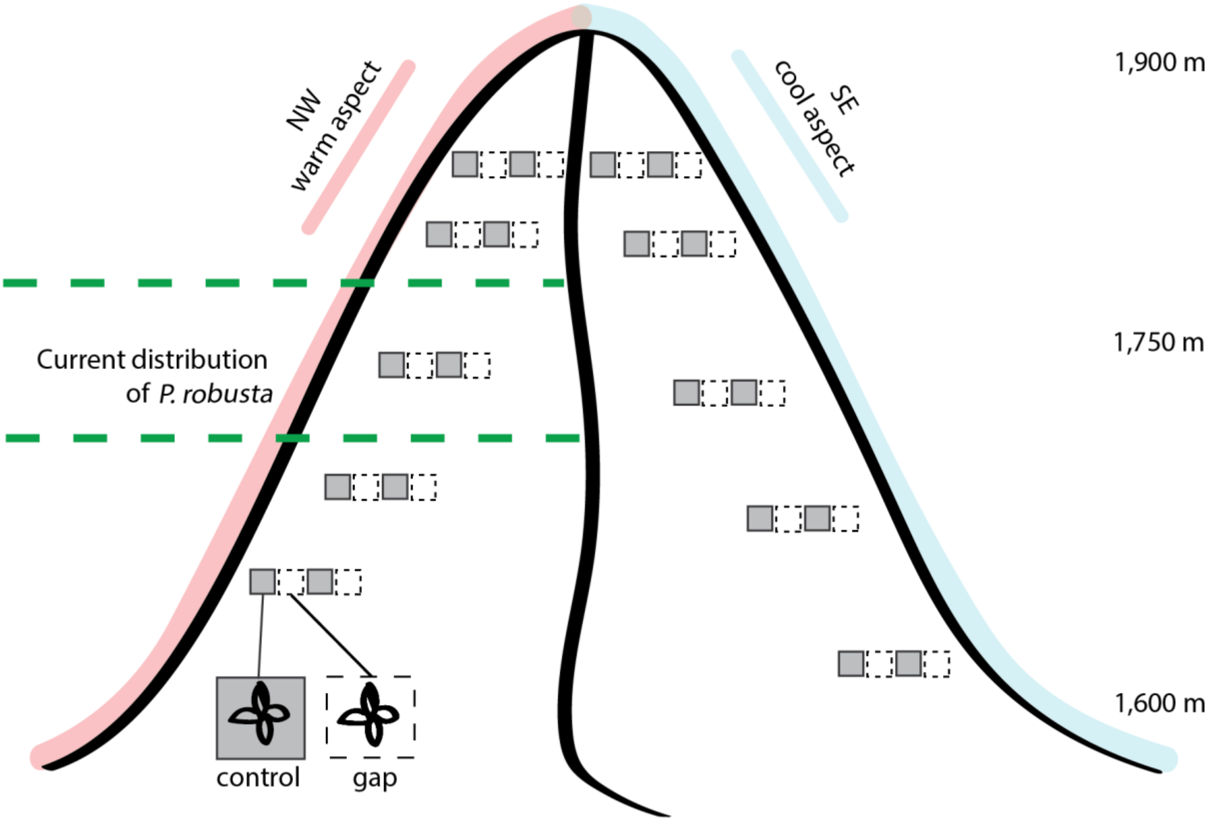

Figure S1: Study design at Mt Hotham, Australia. Ten sites were arranged along a 235 m elevation gradient, with five sites on warm, north-west facing slopes and five sites on cool, south-east facing slopes. *P. robusta* seedlings (n=13) were transplanted into either intact vegetation (control, with neighbouring plants) or vegetation gaps (15cm x 15cm gaps where all vegetation was removed by cutting to ground level). This figure is not representative of the total number of transplant replicates.

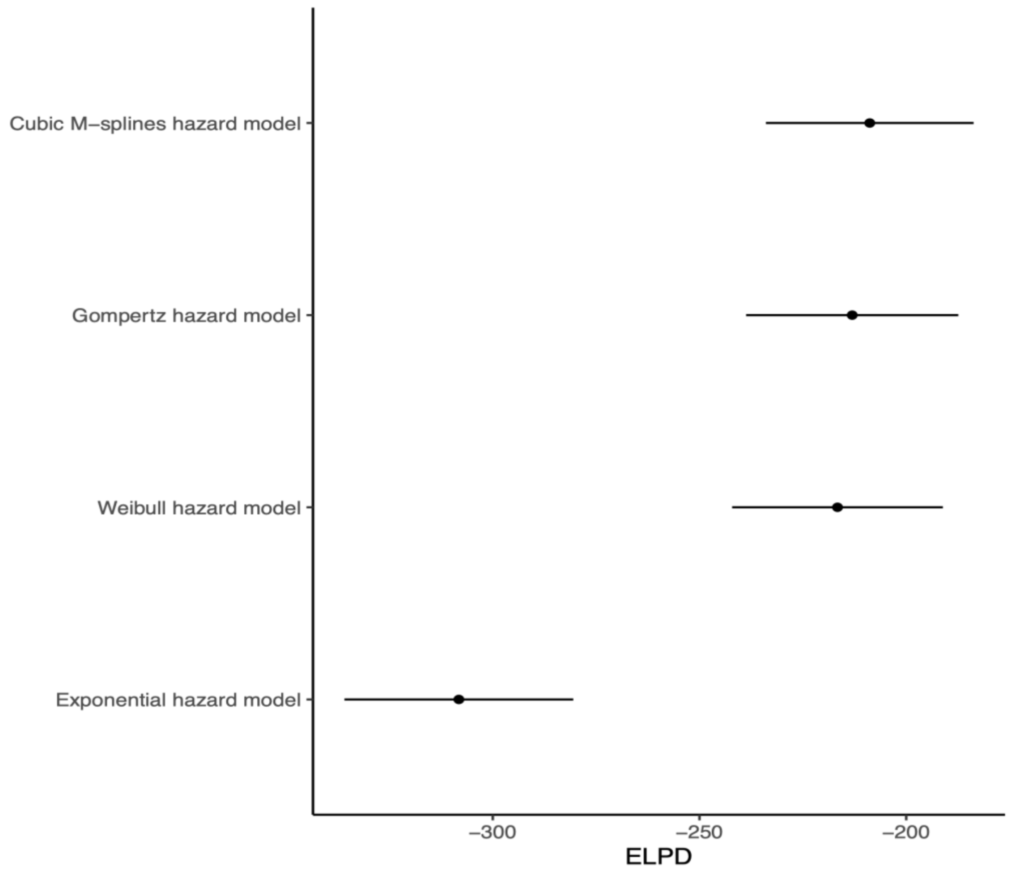

Figure S2: Expected log predicted density for each hazard model. The exponential model was clearly inferior to other models.

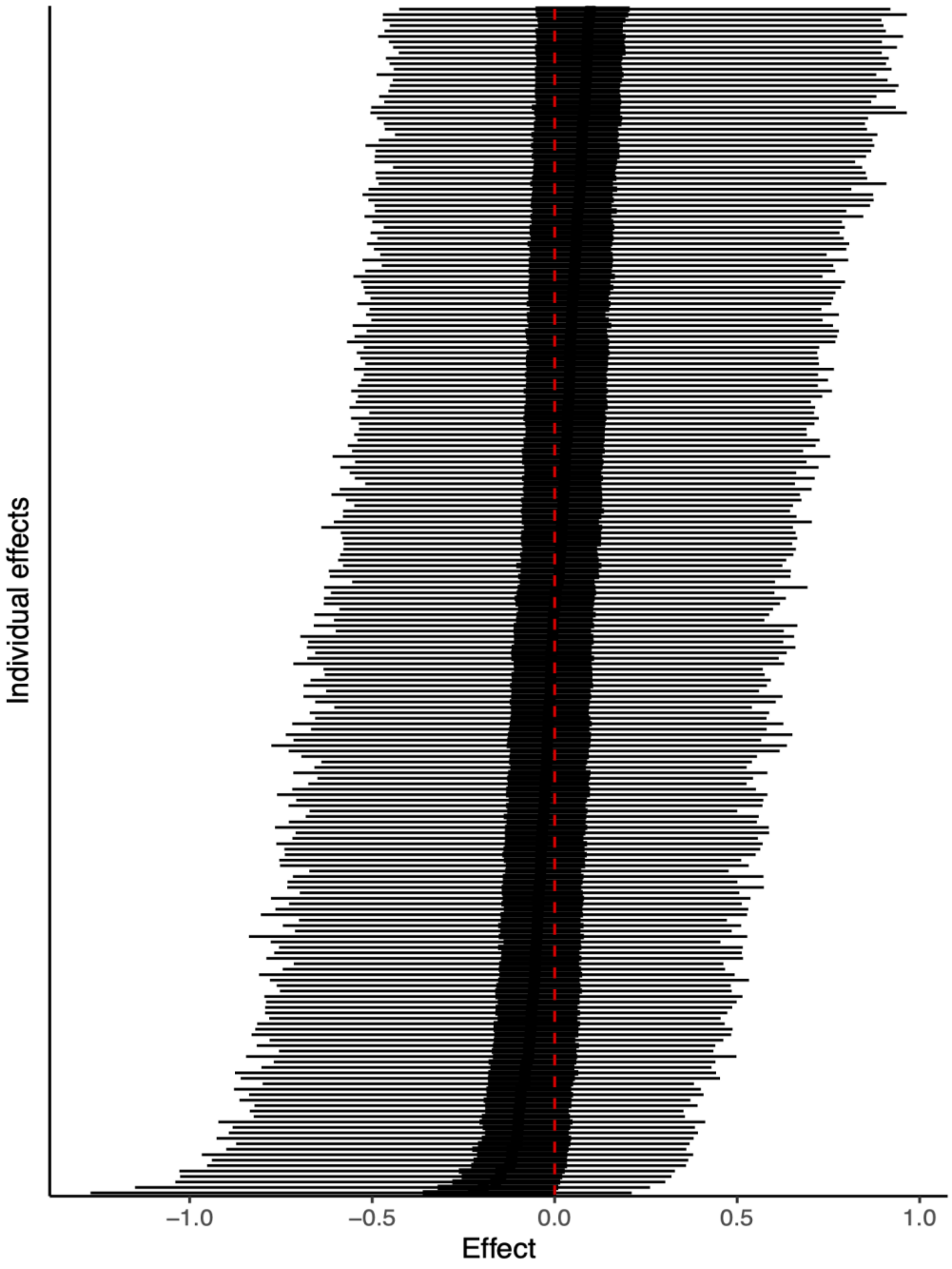

Figure S3: Unexplained residual variation among individuals derived from Cubic M-spline hazard model.

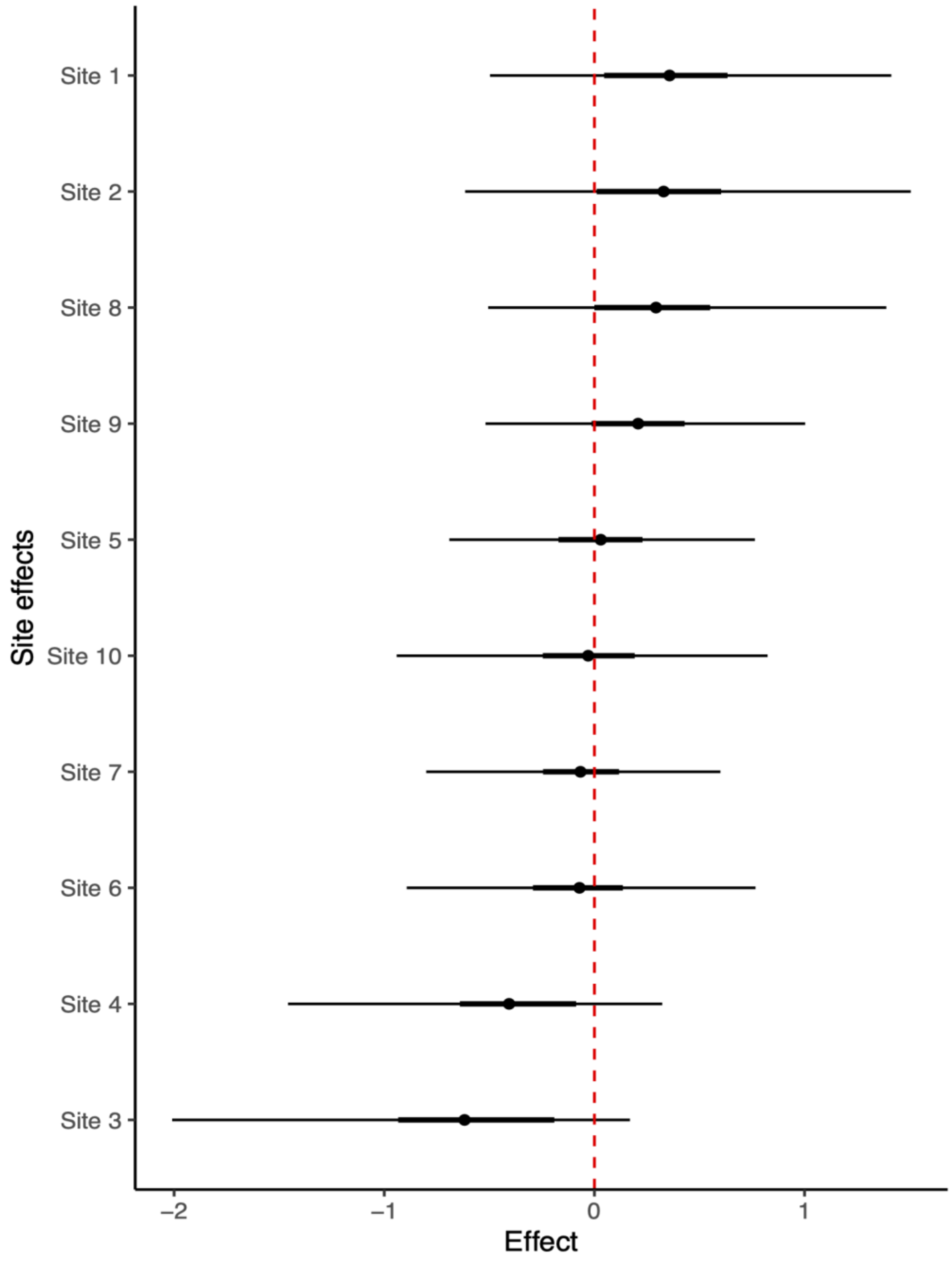

Figure S4: Unexplained residual variation among sites derived from Cubic M-spline hazard model.

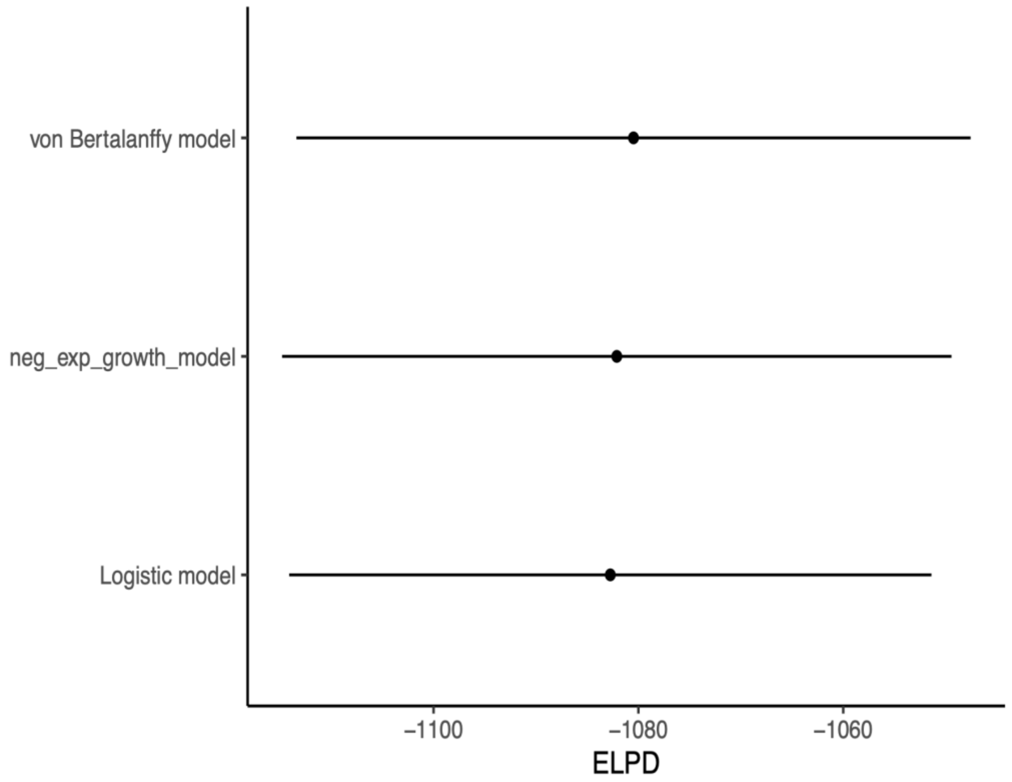

Figure S5: Expected log predicted density for each growth model. All models were similar, though the von Bertalanffy model had slightly higher mean ELPD.

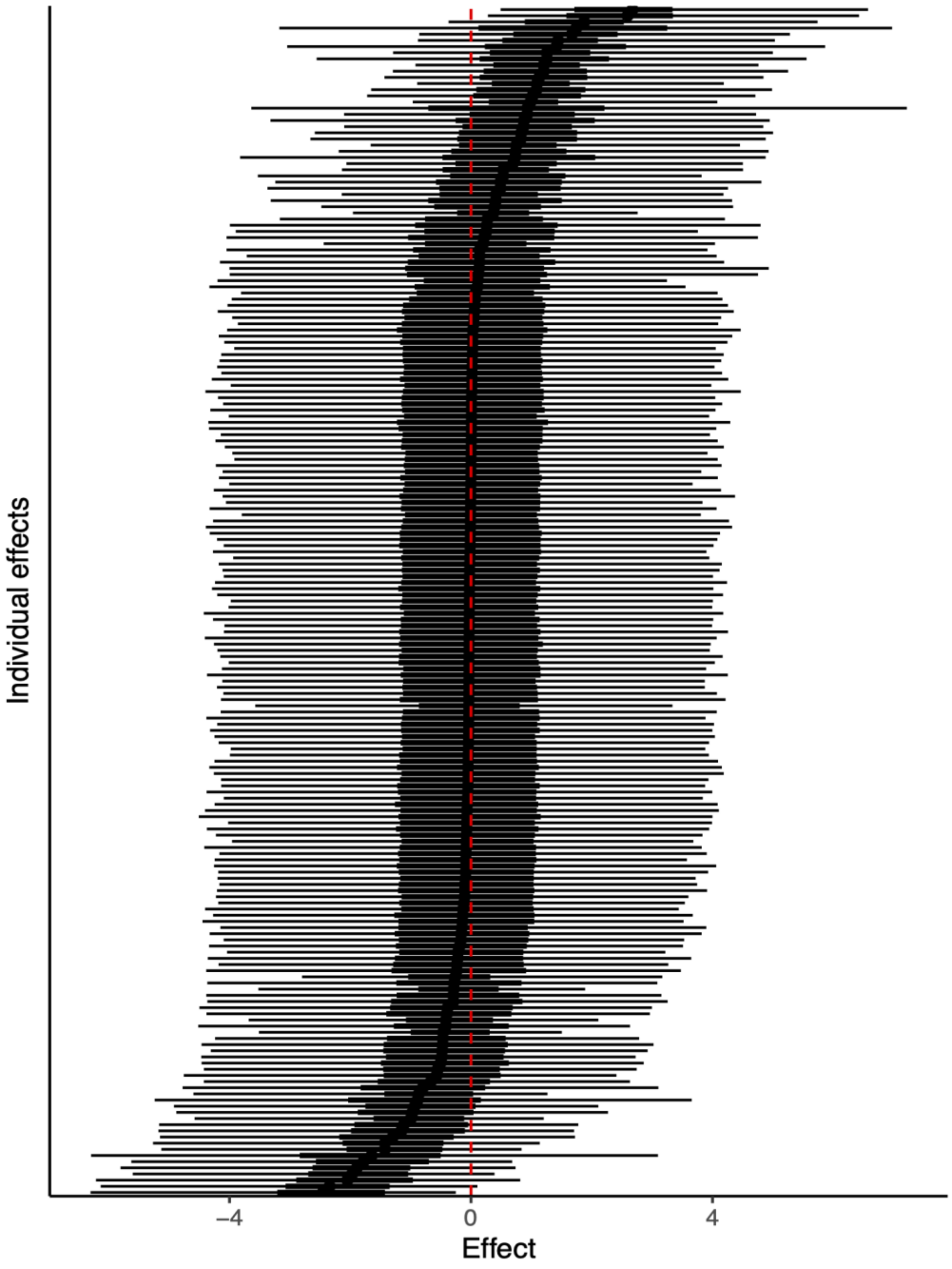

Figure S6: Unexplained residual variation among individuals derived from vb growth model.

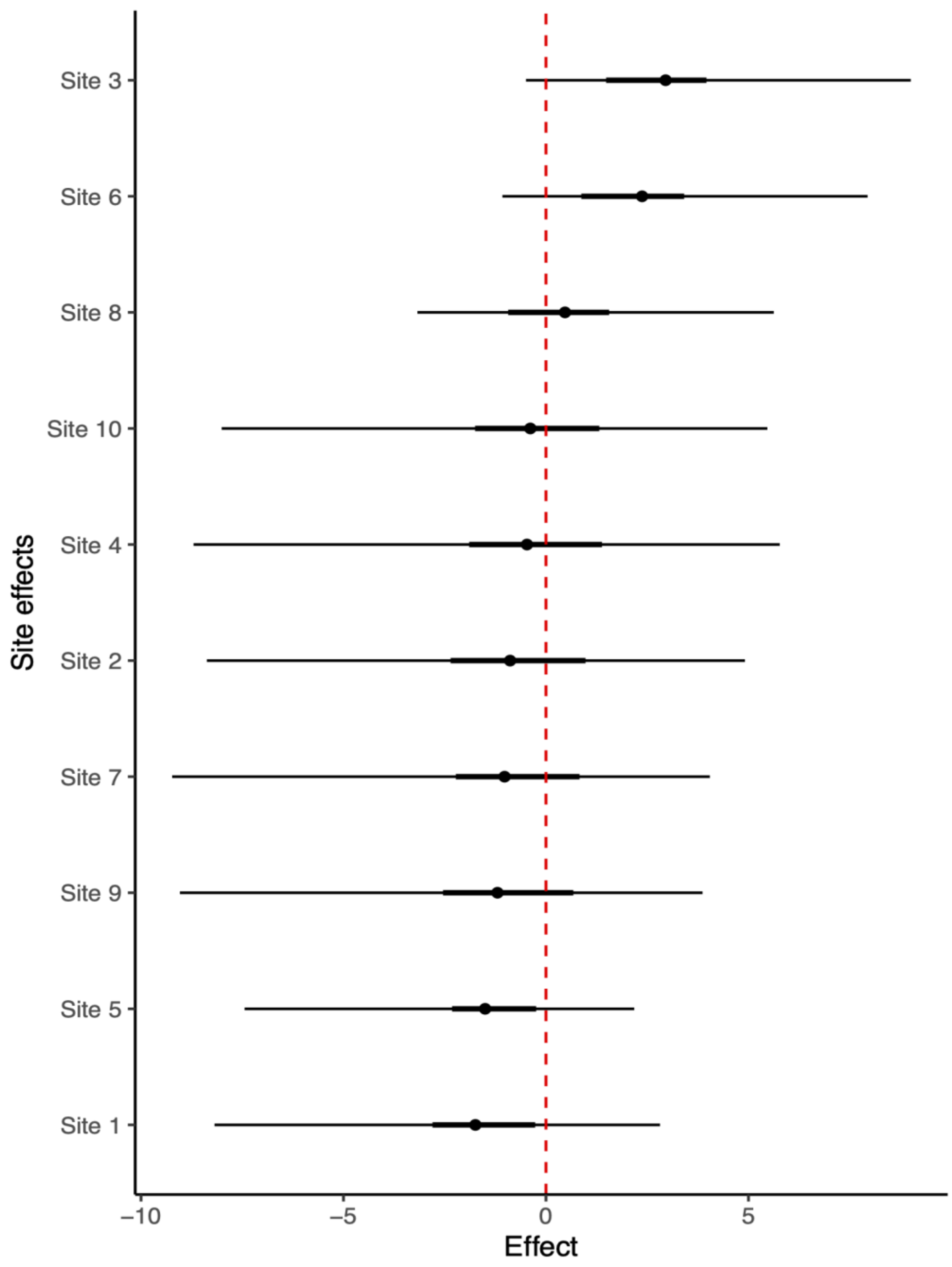

Figure S7: Unexplained residual variation among sites derived from vb growth model.
